# The association between history of appendectomy and gut microbiota composition: a follow-up cross-sectional study

**DOI:** 10.3389/fmicb.2025.1697138

**Published:** 2026-01-07

**Authors:** Matija Hadžić, Paul Hammer, Carsten Krumbiegel, Olga Moskalenko, Andrija Karačić, Daria Hadžić

**Affiliations:** 1 University Hospital Sveti Duh, Zagreb, Croatia; 2 Biomes NGS GmbH, Wildau, Germany; 3 The Gut Microbiome Center, Zagreb, Croatia; 4 University Hospital Center Sestre Milosrdnice, Zagreb, Croatia

**Keywords:** appendectomy, appendix, gut microbiota, gut bacteria, dysbiosis, restorative tendency, postoperative

## Abstract

**Introduction:**

The appendix is thought to act as a regulatory immune organ and gut microbiota reservoir. Although appendectomy is linked to health risks, its impact on the gut microbiota remains understudied.

**Methods:**

This study is conceived as a cross-sectional retrospective follow-up study. Three comparisons were performed on gut microbiota data using self-reported metadata retrieved from a European laboratory’s extensive database. First, subjects with (wA) and without (noA) appendectomy during stool sampling were compared. Second, healthy individuals were selected based on specific criteria by comparing those with (HwA) and without (HnoA) appendectomies. Finally, healthy (HwA) and non-healthy (nHwA) subjects with a history of appendectomy were compared. Due to the study design, the timing and cause of the appendectomy were unknown. Data on confounding factors, such as age, BMI, and sex, were analyzed as covariates. Regarding the gut microbiota, alpha and beta diversity, relative abundance of phyla, genera, and metabolic pathways were compared.

**Results:**

Significant differences were found in the gut microbiota composition and functionality between 2′615 adult subjects who had and 13′103 adults who had not undergone appendectomy (wA vs. noA), but also in confounding factors such as age, sex, and BMI. No significant differences were found in the gut microbiota between the 111 healthy adult subjects and 876 adults who had not undergone appendectomy (HwA vs. HnoA) at the time of stool sampling. Significant differences were found between 111 healthy and 2′504 non-healthy subjects who underwent appendectomy (HwA vs. nHwA). The gut microbiota composition of nHwA differed significantly in beta diversity; it was less diverse (Shannon entropy) and showed a decreased abundance of two genera, *Eubacterium ruminantium* and *Lachnospiraceae* FCS020. The HwA group was found to consume additional portions of vegetables and fruits and sleep longer, but these differences were not significant.

**Conclusion:**

Our study found significant differences in the gut microbiota composition between healthy and non-healthy subjects who underwent appendectomy, but no difference in healthy subjects with or without appendectomy. Further studies are needed to elucidate whether these differences are due to different restorative capacities over time.

## Introduction

The function of the appendix has not yet been fully elucidated (Islam et al., 2025). Contrary to earlier assumptions that the appendix is a vestigial organ (Killinger and Labrie, 2019), evolutionary evidence highlights its pivotal role in intestinal physiology (Laurin et al., 2011). The contemporary hypothesis states that the appendix plays a dual role (Girard-Madoux et al., 2018): one is as a regulatory immune organ, and the other is as a gut microbiota reservoir, which regulates the gut microbiota (Randal Bollinger et al., 2007).

The loss of the appendix in the clinical setting as a consequence of appendectomy has subtle but significant effects on general health (Chen et al., 2020).

The connection between appendectomy and a long list of subsequent disease states has been reported in the scientific literature (Sagor et al., 2025): ileocecal Crohn’s disease (Russel et al., 1997), pyogenic liver abscess (Liao et al., 2016), sarcoidosis in a woman (Sawahata et al., 2017), rheumatoid arthritis in women (Tzeng et al., 2015), chronic kidney disease (Chang et al., 2018), right colon cancer in women (Grobost et al., 1991), Parkinson’s disease (PD) (Nakahara et al., 2023), *Clostridium difficile* infection, and mental disorders such as depression, anxiety, and bipolar disorder (Ekström et al., 2020).

The link between appendectomy and all the aforementioned disease states is supposedly the ensuing dysbiosis and low-grade systemic inflammation (Sánchez-Alcoholado et al., 2020; Vitetta et al., 2019). However, this association has not been well documented, especially not in a causative manner. A significant impact of appendectomy on the gut microbiota seems plausible; however, the direction and magnitude, as well as the permanence of the effect, are only partially documented.

A gap in knowledge was detected in the scientific literature, since only a small number of studies have investigated this topic. The only study that intentionally monitored the questionable effect of appendectomy observed a loss of short-chain fatty acid (SCFA)-producing genera, especially butyrate, such as *Roseburia, Barnesiella, Butyricicoccus, Butyricimonas,* and *Odoribacter,* in favor of an increase in the gut fungal community (Cai et al., 2021), with a certain restorative tendency with time. Another study found that incidental prophylactic appendectomies led to a decrease in the richness and diversity of the gut microbiota, especially the genera *Odoribacter, Butyricimonas, Bilophila,* and *Faecalibacterium,* which were associated with impaired insulin regulation in this patient population (Sánchez-Alcoholado et al., 2020). Other authors have concluded that post-appendectomy dysbiosis promotes carcinogenesis/colorectal tumorigenesis and leads to an impaired intestinal barrier function (Chen et al., 2020).

To contribute more data on the association between a history of appendectomy and gut microbiota composition, an observational cross-sectional study based on secondary data analysis from a database of 16S rRNA amplicon-sequenced fecal samples was performed. The study design and data analysis plan were based on those of an earlier study by Cai et al. (2021). Therefore, this study was conceived as a follow-up study. Aware of the suboptimal data source, since data on the timing and cause of appendectomy are lacking, the aim of the study was to compare the gut microbiota composition and functionality of healthy individuals with and without a history of appendectomy to determine whether a history of appendectomy could differentiate taxonomic and functional profiles significantly between the two groups. Differences in diversity, relative abundances of taxonomic groups, and relative abundances of metabolic pathways and functional modules were statistically analyzed. Based on the original study by Cai et al. (2021), the hypothesis was that significant differences in taxonomic profiles in healthy subjects with and without appendectomy were to be found: a significant shift in *β*-diversity and significant differences in the abundances of pathobionts such as *Escherichia-Shigella* and *Klebsiella,* and SCFA-producing genera such as *Roseburia*, *Barnesiella*, and *Butyricicoccus.* By assessing the significant differences between the two groups, this study aims to provide more data on the association between appendectomy and dysbiosis and thereby shed more light on the specific health risks of appendectomy.

## Study population and methods

### Data

The data utilized in this study were retrieved from the data bank of Biomes NGS Ltd. (Wildau, Germany). This company has been offering self-testing for the gut microbiota since 2017. Only data with an explicit indication of consent for scientific use by customers were included in the respective data bank. This data bank comprises more than 50′000 samples associated with individual lifestyle information from individuals mostly from Europe. For this study, samples included in the data bank from December 2017 to December 2024 were analyzed. Lifestyle information was provided by the participants through an online self-report questionnaire. Although the scale of the data bank is extensive due to the low-threshold nature of the self-report questionnaire, the respective lifestyle data were not from a clinical survey with medical supervision, therefore lacking any clinical validation. Both the sampling process and questionnaire completion were conducted by the customers themselves. Because the self-test is a lifestyle product, the customer’s information has not been further verified by a medical doctor or a similar expert.

The data bank contained the 16S ribosomal DNA (rDNA)-sequenced microbiota profiles of the stool samples. Microbial DNA was analyzed using next-generation sequencing (NGS) of the 16S rRNA gene as an amplicon, as described in the Methods section. The microbiota profile was composed of normalized counts per taxonomic level (kingdom, phylum, class, order, family, genus, and species).

The lifestyle questionnaire consisted of 99 questions (Supplementary File S1: Lifestyle questionnaire). It assesses anthropometric measures, sociodemographic data, current health status, medical history, bowel movement, food intake via a food frequency questionnaire, sleep, physical activity, and other lifestyle factors. One question asked the individual to confirm whether “his appendix was reformed,” and one could answer either positively or negatively (“yes”/“no”). Any answer to this specific question was the principal inclusion criterion. The subjects were not asked about the time since appendectomy or the pathohistological diagnosis of the removed appendix.

The data bank was sourced for adequate study subjects by a competent bioinformatician who had given explicit consent for their data to be used for scientific purposes and had provided answers to the entire lifestyle questionnaire. In addition to data on the gut microbiota profiles, data on age, sex, and BMI were included in the data analysis as potential confounding factors to minimize possible biases.

### Study design

The study design was conceived as a follow-up to the original study by Cai et al. (2021). An observational cross-sectional study was conducted based on the secondary data analysis of the large-scale data bank described in the previous section. The study size was not evaluated before data retrieval.

The study was conducted in accordance with the Strengthening the Organization and Reporting of Microbiome Studies (STORMS) (Mirzayi et al., 2021) and Strengthening the Reporting of Observational Studies in Epidemiology (STROBE) (Cuschieri, 2019) guidelines.

### Participants

In this study, data from samples with explicit consent for scientific use and a completed lifestyle questionnaire were included, specifically regarding the history of appendectomy. Three data comparisons were performed.

First, the samples were sorted into two groups based on their response to the appendectomy question: samples of individuals who reported having undergone appendectomy in the online self-report questionnaire (answered “yes”) (group wA) and samples of individuals who did not (answered “no”) (group noA) as controls. The exclusion criteria were age <18 years and a history of gastrointestinal surgery besides appendectomy.

To further investigate the differences between those with and without appendectomy, healthy individuals were singled out in a distinct cohort (groups HwA and HnoA). Healthy individuals were defined fundamentally as those who did not self-report any chronic illnesses, complaints, or medications during questionnaire completion.

Additionally, a third data comparison was conducted among individuals who had undergone an appendectomy. They were subdivided into two groups: healthy subjects (HwA) and non-healthy subjects (nHwA).

In the healthy individual subgroup, the subjects met the following exclusion criteria:

- No chronic illnesses known to affect the gut microbiota.
- Diabetes, IBD, IBS, autoimmune diseases, metabolic disorders, oncological diseases, mental illnesses.
- No chronic medication.
- e.g., proton pump inhibitors, antidepressants.
- No gluten allergy and no gluten-free diet.
- No abdominal complaints (bloating, nausea, and colics).
- No daily alcohol consumption.
- Normal stool consistency (Bristol scale 3, 4, 5).
- No use of antibiotics within the last 90 days.
- No use of probiotics within the last 90 days.

### Methods (sample collection, amplicon sequencing, and bioinformatic analyses)

Gut microbiome analysis of all fecal samples was performed in the same laboratory, so bias resulting from the sequencing process and bioinformatic analysis could be eliminated. For stool sampling, cotton swabs were used. Individuals were instructed to swab the amount of pinhead fecal material from toilet paper.

### Sample storage and lysis

Upon arrival at the laboratory in vials containing 1,000 μL DNA-stabilizing buffer solution, all collected stool samples were stored at −20 °C until use. For the lysis process, the samples were defrosted and centrifuged at ~3,000–5,000 × g for 3–5 min at room temperature. Afterwards, warmed PW buffer from the QIAamp 96 PowerFecal QIAcube HT Kit (Qiagen, Hilden, Germany) was added to each sample.

### Extraction of stool samples

For DNA extraction, the QIAamp 96 PowerFecal QIAcube HT Kit (Qiagen, Hilden, Germany) was established on our liquid handling systems [Hamilton StarLine (Hamilton Company, Reno, Nevada, United States) & Tecan EVO (Tecan Group Ltd., Männedorf, Switzerland)] using a vacuum chamber as well as a high-pressure chamber. The extracted gDNA was stored at −20 °C until use.

### Library preparation for sequencing with the Illumina MiSeq System

Library preparation followed the manual “16S Metagenomic Sequencing Library Preparation—Preparing 16S Ribosomal RNA Gene Amplicons for the Illumina MiSeq System” (Illumina, San Diego, CA, United States). The mastermix reagents utilized for target and library amplification were sourced from New England BioLabs (New England BioLabs Ltd., Ipswich, Massachusetts, United States), and the 16S rRNA V3V4 primer from Eurofins Genomics (Eurofins Scientific SE, Luxembourg City, Luxembourg). For normalization of all samples, a fluorescent dye and the Biotek Synergy HTX Plate Reader (Agilent Technologies, Santa Clara, CA, United States) were used to measure DNA concentrations and calculate the necessary dilution volume per sample. To ensure high throughput, all the steps, from the first amplification to library pooling, are nearly fully automated in the Biomes NGS Ltd. laboratory using liquid handling systems, e.g., the Hamilton StarLine (Hamilton Company, Reno, Nevada, United States). Hence, the laboratory can process 96 and 192 samples simultaneously, and the normalization also works for up to 288 samples. Library denaturing and MiSeq sample loading were performed manually.

### Processing sequence reads

The determined paired-end reads were filtered as follows: First, the forward/reverse reads were merged using PANDAseq (Masella et al., 2012). This was followed by an alignment using BLASTn (Altschul et al., 1990) against the SILVA rRNA database (version: 138.1) (Quast et al., 2013). Subsequently, filtering was performed. For further analysis, the minimum requirement was at least 10,000 assigned reads per sample. At different taxonomic levels, different identity thresholds (phylum: 75.0%; class: 78.5%; order: 82.0%; family: 86.5%; genus: 94.5%; and species: 97.0%) (Yarza et al., 2014) were applied. The sequences were clustered according to their similarity (97%) using CD-HIT (Li and Godzik, 2006; Fu et al., 2012). Biologically normalized abundances were calculated from clustered reference sequences using the PICRUSt2 pipeline (Douglas et al., 2020). The final output of the entire process is a table of biologically normalized counts per taxonomic level. The PICRUSt2 pipeline was used to determine the available pathways according to the MetaCyc library for each sample by using a predictive model (Caspi et al., 2020). The abundances of the identified pathways were calculated based on the respective gene families.

Alpha diversity was based on rarefied raw counts. The alpha diversity metrics—Shannon entropy (Herrera et al., 2023) and the Chao index (Chao and Shen, 2003)—were calculated based on the ASV table, and the Pielou evenness index (Pielou, 1966) was computed based on species abundance. Shannon diversity was calculated using QIIME2 (Bolyen et al., 2019), whereas Chao1 and Pielou’s evenness were computed using the scikit-bio (skbio) v0.5.6 package (Pedregosa et al., 2011). Further analysis was performed with custom Python scripts using the pandas 2.2.3 (Mckinney, 2011), NumPy 2.2.5 (Harris et al., 2020), scikit-bio (Pedregosa et al., 2011), and SciPy v1.11.1 (Virtanen et al., 2020) libraries.

### Statistical analysis

The data scientists used various statistical tests to detect significant differences between the different groups. To determine whether the numerical values of the analyzed microbiota and lifestyle data followed a normal distribution, the D’Agostino-Pearson test was used (D’Agostino, 1971; D’Agostino and Pearson, 1973). If certain relative taxa and pathway abundances were not normally distributed, the median, interquartile range (IQR), and minimal and maximum values were calculated. A Student’s *t*-test was applied to normally distributed samples, and the Mann–Whitney U test was applied to non-normally distributed samples. Because the median of certain taxa reached near-zero values, the respective taxa’s abundance was equated to zero to facilitate further analysis. Unspecified genera were excluded from data analysis, as well as mean values below 0.1%. For Venn diagrams, the median of a specific OTU in the group had to be above 0.1, and the OTU had to be present in at least 5% of the samples of the respective group.

The significance of differences in the diversity indices and individual taxa was tested using a non-parametric Wilcoxon rank-sum test for the two groups with Benjamini–Hochberg corrections (Benjamini and Hochberg, 1995) using the Scipy v1.11.1 package (Virtanen et al., 2020). Beta diversity was measured using the Bray–Curtis distance, and significance was determined using PERMANOVA with 9,999 permutations implemented using the Python package scikit-bio (Pedregosa et al., 2011). Correlations between bacterial taxa were computed and tested using the Wilcoxon rank-sum test, and the Benjamini-Hochberg correction for false positivity was applied. Finally, the results were visualized using a custom Python script based on Seaborn and matplotlib or VennDiagram (matplotlib_venn). All analyses were performed using Python v3.11.8.

To determine correlations between individual lifestyle categorical data, the chi-square test of independence was used. A *p*-value of ≤0.05 was considered significant for all tests used.

To evaluate whether a history of appendectomy independently influenced gut microbiota diversity and composition, multivariate regression models were constructed for each outcome measure. Alpha-diversity indices (Shannon diversity and Pielou evenness) and the relative abundance of Lentisphaerae were analyzed using linear regression models, whereas beta-diversity (Bray–Curtis dissimilarity) was assessed using permutational multivariate analysis of variance (PERMANOVA, adonis2 function, vegan package, R). In all models, age, BMI, and appendectomy status (coded as categorical variables) were included as covariates. Analyses were performed separately for the total study population and a subset of participants without chronic diseases (healthy population). Model fit was evaluated using the coefficient of determination (*R*^2^), and statistical significance was assessed at *p* < 0.05.

## Results

### Characteristics of the study populations

**Figure 1:**
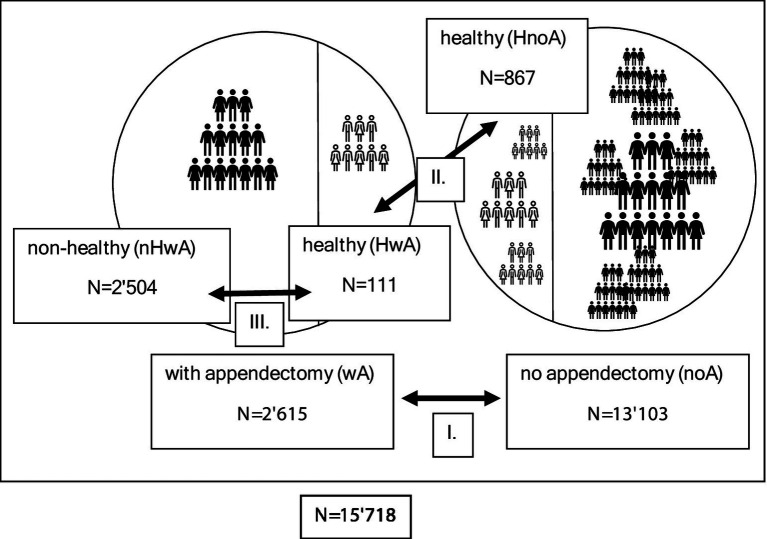
Graphic presentation of the three data comparisons conducted in this study. I: comparison between individuals with and without appendectomy. II: comparison between healthy individuals with and without appendectomy. III: comparison between healthy and non-healthy individuals with appendectomy (*N* indicates the number of subjects in each group).

The total study population included 15′718 individuals, and three data comparisons were run in this cohort (Figure 1).

The group after appendectomy (wA) numbered 2′615 individuals, and the group without appendectomy (noA) 13′103 individuals. There were significant differences in age, BMI, and sex between the wA and noA groups (Table 1). The BMI of wA was greater than that of noA (22.15 ± 1.7 25 kg/m^2^ vs. 22.04 ± 1.72 25 kg/m^2^; *p* < 0.01), but both were within normal limits (18.5 ≤ BMI < 25 kg/m^2^). For the purpose of the follow-up study, 987 healthy wA and noA individuals were singled into two groups: 111 healthy individuals after appendectomy (HwA) and 867 healthy individuals without appendectomy (HnoA) (Table 2). Subjects from the HwA group were significantly older than those from the HnoA group (46.94 ± 12.22 compared to 41.84 ± 12.04 years). For additional data comparison, the wA group was subdivided into two groups: the gut microbiota of the 111 healthy individuals after appendectomy (HwA) was compared to those of 2.504 unhealthy individuals after appendectomy (nHwA), subjects who, besides a history of appendectomy, also reported chronic illnesses or taking chronic medication (Table 3). Subjects from the nHwA group were significantly older than those from the HwA group (50.37 ± 14.16 vs. 46.94 ± 12.22, *p* < 0.01) and predominantly female (71.5%).

**Table 1:** Total study population (*N* = 15.718).

	wA (*N* = 2.615)	noA (*N* = 13.103)	*p*-value
Age (years, mean SD)	50.22 ± 14.1	42.85 ± 13.33	<0.01^1^
Sex (male/female)	767/1848	4381/8722	<0.01^2^
BMI (kg/m^2^, SD)	22.15 ± 1.7	22.04 ± 1.72	<0.01^1^

*N*, number of subjects; SD, standard deviation; BMI, body mass index; wA, subjects with appendectomy; noA, subjects without appendectomy; ^1^Mann–Whitney test, ^2^Chi-square test.

**Table 2:** Healthy study population (*N* = 987).

	HwA (*N* = 111)	HnoA (*N* = 876)	*p*-value
Age (years, mean SD)	46.94 ± 12.22	41.84 ± 12.04	<0.01^1^
Sex (male/female)	54/57	426/450	0.998^2^
BMI (kg/m^2^, SD)	22.29 ± 1.8	22.25 ± 1.68	0.685^1^

*N*, number of subjects; SD, standard deviation; BMI, body mass index; HwA, healthy subjects with appendectomy; HnoA, healthy subjects without appendectomy; ^1^Mann–Whitney test, ^2^Chi-square test.

**Table 3:** Appendectomy study population (*N* = 2.615).

	HwA (*N* = 111)	nHwA (*N* = 2.504)	*p*-value
Age (years, mean SD)	46.94 ± 12.22	50.37 ± 14.16	0.011^1^
Sex (male/female)	54/57	713/1791	<0.01^2^
BMI (kg/m^2^, SD)	22.29 ± 1.8	22.15 ± 1.7	0.330^1^

*N*, number of subjects; SD, standard deviation; BMI, body mass index; HwA, healthy subjects with appendectomy; nHwA, non-healthy subjects with appendectomy; ^1^Mann–Whitney test, ^2^Chi-square test.

### Gut microbiota alterations

**Figure 2:**
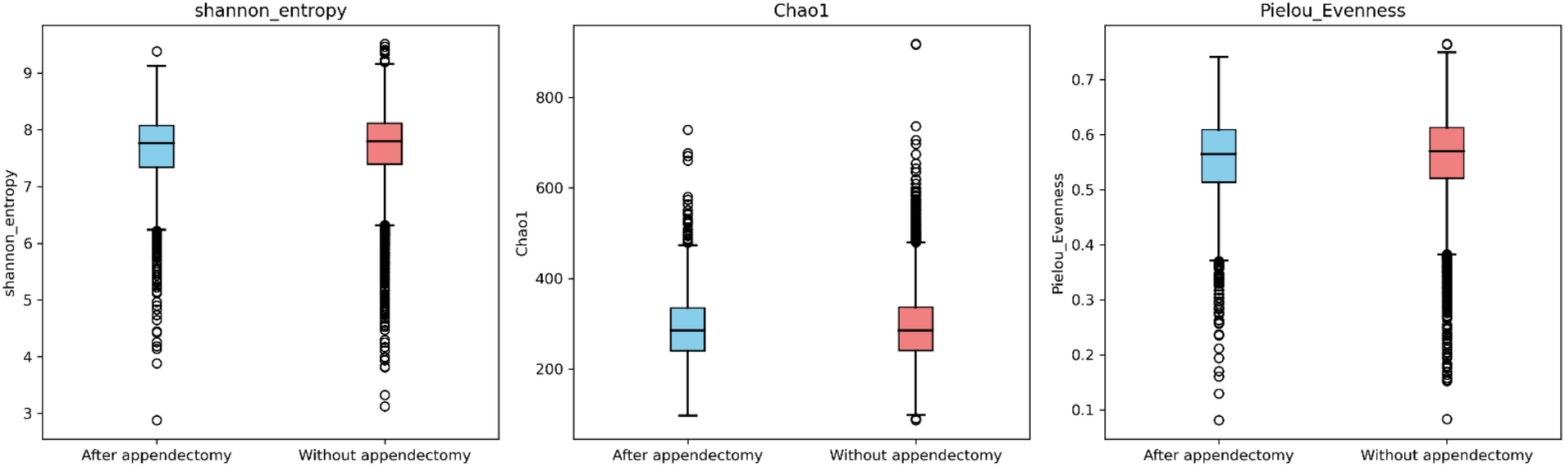
Alpha diversity was estimated by Shannon diversity, Chao1 richness (observed OTUs), and Pielou’s evenness in the total study population.

Alpha diversity was evaluated using Shannon entropy, Chao1 richness, and Pielou evenness metrics. Statistically significant differences were detected between the wA and noA groups regarding the Shannon entropy (*p* = 0.002) and Pielou evenness (*p* = 0.001) metrics, but not between the HwA and HnoA groups. The wA group had lower median values for all three indices (Figure 2).

**Figure 3:**
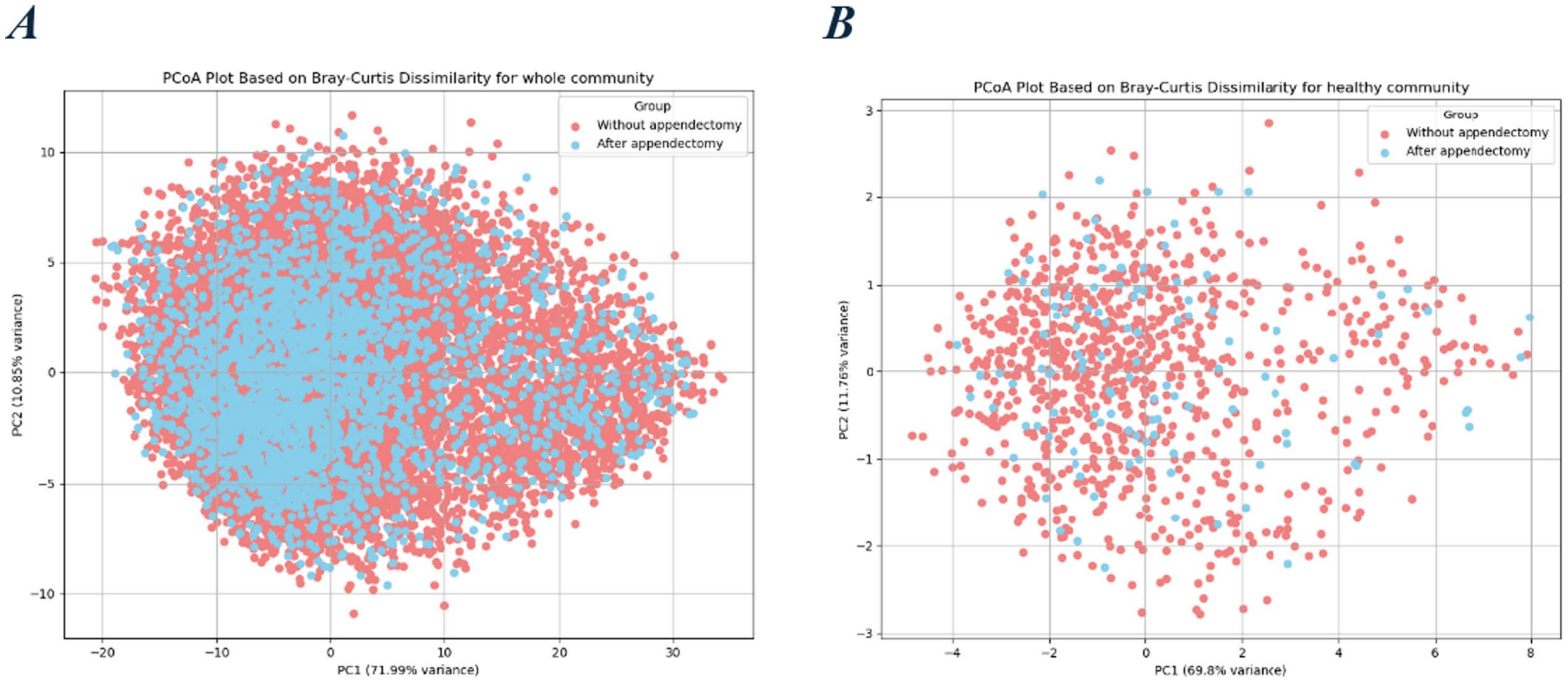
Beta diversity: distribution of Bray–Curtis dissimilarity values among samples and Principal Coordinates Analysis (PCoA). **(A)** Total study population, **(B)** healthy study population.

Beta diversity analysis revealed significant differences in the gut microbiota composition between the wA and noA groups (PERMANOVA *F* = 10.23, *p* = 0.001), but not between the HwA and HnoA groups (PERMANOVA *F* = 1.09; *p* = 0.317) (Figure 3).

**Figure 4:**
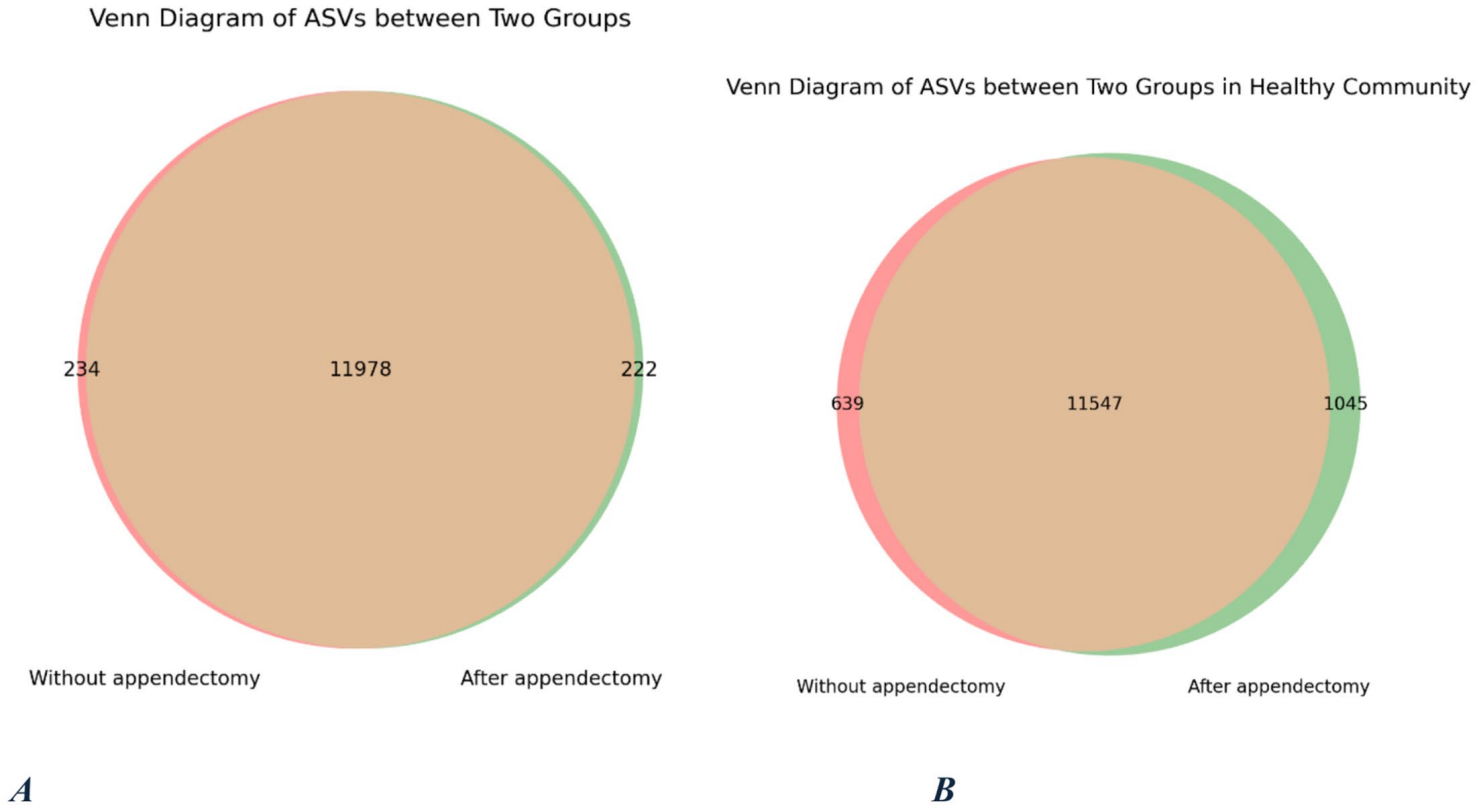
Venn diagram of OTUs shared by and exclusive to the two groups. OTU, operational taxonomic unit. **(A)** Total study population, **(B)** healthy study population.

The Venn diagram shows that the wA and noA groups shared 11′978 universal OUT (Figure 4). A total of 222 OUT were present in the wA group but not detected in the noA samples. The HwA and HnoA groups shared 11′547 universal OUT. In the HwA group, 1,045 OUT were present but not detected in the HnoA samples.

After correcting for false positives, taxonomic differences were found to be scarce. Only the phylum Lentisphaerae was reduced in the total study population comparison (q < 0.01), and at the genus level, no significant differences were detected.

In the total study population, significant differences were observed in 88 metabolic pathways between the two groups. In the healthy study population, on the other hand, significant differences were detected only in the abundances of six pathways, but all of those were found to be likely falsely positive.

**Figure 5:**
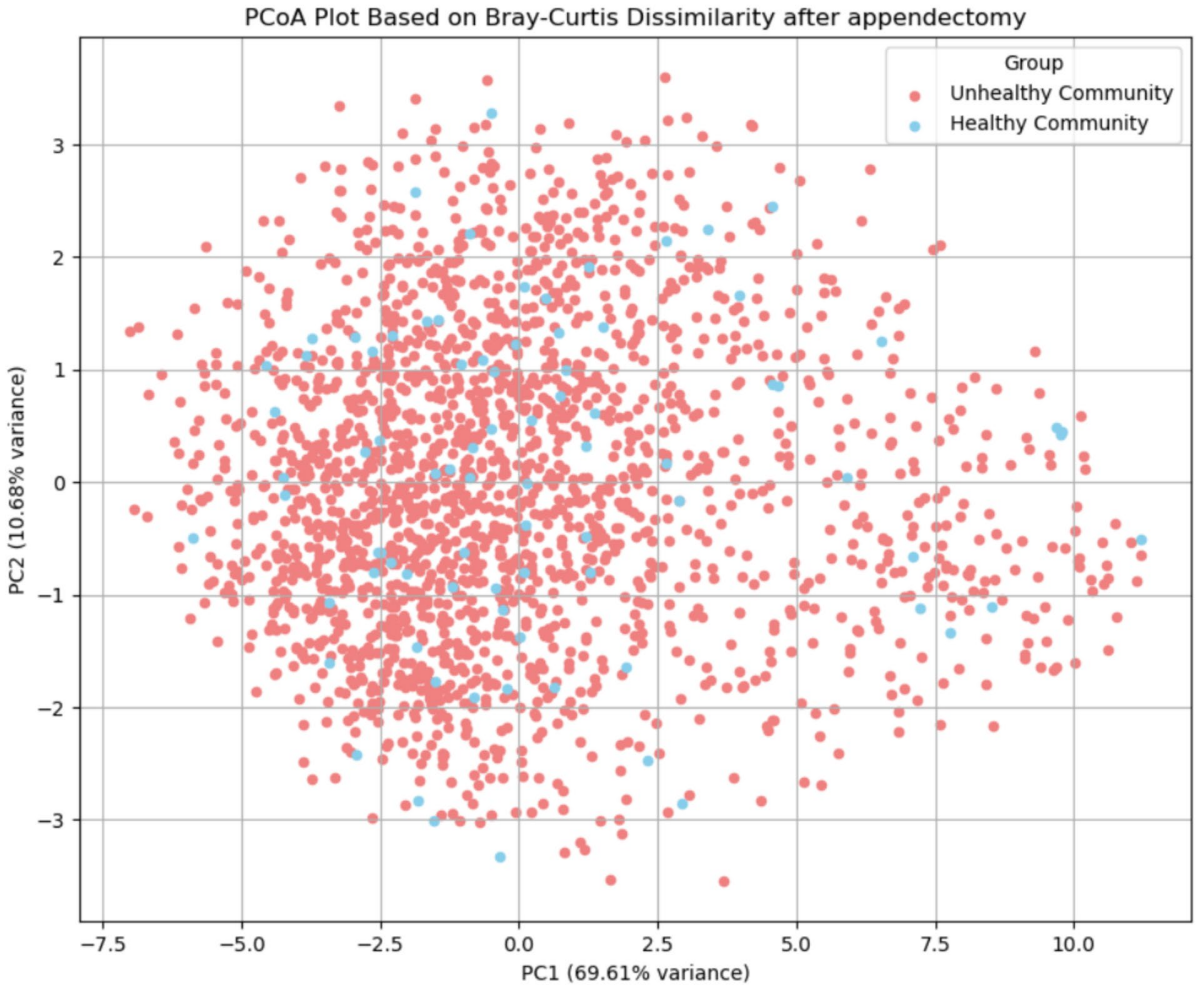
Beta diversity: distribution of Bray–Curtis dissimilarity values among samples with appendectomy and Principal Coordinates Analysis (PCoA).

The comparison of the gut microbiota among subjects who underwent appendectomy revealed significant differences in alpha diversity and beta diversity. The median values of all three alpha diversity metrics were lower in the nHwA group (Table 4), indicating lower diversity of the gut microbiota in non-healthy subjects who underwent appendectomy. The beta diversity differed significantly as well (PERMANOVA *F* = 2.47, *p* = 0.004) (Figure 5). Regarding significant differences in the abundance of taxonomic groups, only the genera *Eubacterium ruminantium* and *Lachnospiraceae FCS020* were found to be significant even after false positivity testing (FDR < 0.032). Although 23 metabolic pathways were found to be significantly altered, the difference appeared to be a false positive (FDR > 0.343).

**Table 4:** Median values of alpha diversity metrics in the appendectomy study population.

	nHwA (*N* = 2.504)	HwA (*N* = 111)	*p*-value
Shannon entropy	7.76	7.92	*p* < 0.01^1^
Chao1 richness	285	288.25	0.982^1^
Pielou’s evenness	0.566	0.567	0.641^1^

*N*, number of subjects; HwA, healthy subjects with appendectomy; nHwA, non-healthy subjects with appendectomy; ^1^non-parametric Wilcoxon rank-sum test.

In the total population (Table 5), multivariable regression models demonstrated that age was significantly associated with several microbiota diversity measures, including Shannon diversity (*R*^2^ = 0.014, *p* < 0.001), Lentisphaerae abundance (*R*^2^ = 0.002, p < 0.001), and beta diversity based on Bray–Curtis dissimilarity (PERMANOVA *p* = 0.001). A history of appendectomy was significantly associated with these outcomes (*p* < 0.001 for Shannon diversity, Lentisphaerae, and PERMANOVA), whereas BMI showed no significant association (all *p* > 0.4).

**Table 5:** Multivariable regression analysis of gut microbiota diversity and composition in the total population (*n* = 15,718).

Outcome	*R* ^2^	*p* (Age)	*p* (BMI)	*p* (Appendectomy)	*n*
Shannon_entropy	0.014	<0.001	0.513	<0.001	15,718
Pielou_Evenness	0.001	0.478	0.884	<0.001	15,718
Lentisphaerae	0.002	<0.001	0.442	<0.001	15,718
Permanova (Bray)	0.000651	0.001	0.001	0.001	15,718

*R*^2^, coefficient of determination; *p* (Age), *p*-value for age; *p* (BMI), *p*-value for body mass index; *p* (Appendectomy), *p*-value for appendectomy status; *n*, number of participants; PERMANOVA (Bray), permutational multivariate analysis of variance using Bray–Curtis dissimilarity; BMI, body mass index (kg/m^2^).

Within the healthy population subset (Table 6), age remained a significant predictor of the Shannon diversity index (*R*^2^ = 0.021, *p* < 0.001) and Lentisphaerae abundance (*R*^2^ = 0.008, *p* = 0.019). In this group, BMI showed a weak association only with beta diversity (PERMANOVA, *p* = 0.008), whereas appendectomy status was not significantly associated with any outcome measure (all *p* > 0.27).

**Table 6:** Multivariable regression analysis of gut microbiota diversity and composition in the healthy population (*n* = 987).

Outcome	*R* ^2^	*p* (Age)	*p* (BMI)	*p* (Appendectomy)	*n*
Shannon_entropy	0.021	<0.001	0.155	0.551	987
Pielou_Evenness	0.002	0.505	0.232	0.619	987
Lentisphaerae	0.008	0.019	0.107	0.579	987
Permanova (Bray)	0.001109	0.001	0.008	0.276	987

*R*^2^, coefficient of determination; *p* (Age), *p*-value for age; *p* (BMI), *p*-value for body mass index; *p* (Appendectomy), *p*-value for appendectomy status; *n*, number of participants; PERMANOVA (Bray), permutational multivariate analysis of variance using Bray–Curtis dissimilarity; BMI, body mass index (kg/m^2^).

Overall, age consistently explained the largest proportion of variance across alpha- and beta-diversity measures, whereas appendectomy was significantly associated with gut microbiota composition only in the total population and not in healthy participants.

**Figure 6:**
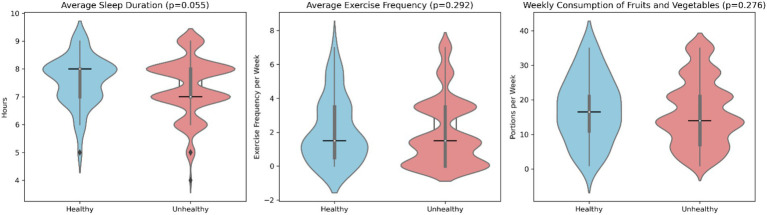
Data on selected lifestyle habits in the appendectomy population (healthy: HwA; unhealthy: nHwA).

To compare the lifestyles of HwA and nHwA, their self-reported metadata on three habits were compared: the reported weekly number of vegetable and fruit portions as an indicator of fiber consumption, the reported number of weekly training sessions, and the reported number of hours of daily sleep at the time of stool sampling (Figure 6). No information was available regarding the temporality of those three habits. Most HwAs were found to eat more portions of vegetables and fruits per week (16 vs. 14 portions) and sleep for almost 1 h at a median of 8 vs. 7 h. The difference in sleep duration was statistically significant (*p* = 0.055).

## Discussion

This study was conceived as a cross-sectional follow-up study to the longitudinal study by Cai et al. (2021). Significant differences were found between healthy and non-healthy subjects who underwent appendectomy. Although there were great differences in the study design and data retrieval, similar to the original study, in the healthy population, no significant differences were detected regarding alpha diversity, as measured with three different metrics. At the genus level, we could not confirm the original study’s finding regarding significant decreases in the abundances of SCFA-producing genera (e.g., *Roseburia*, *Barnesiella*, *Odoribacter*, *Butyricicoccus*). Even the functional modules of SCFA production revealed no significant differences. In our study, the marked impact of appendectomy on the gut microbiota, as observed in the original study, was not replicated. However, the authors of the original study did demonstrate a restorative tendency of the gut microbiota composition after an appendectomy with time. This restorative tendency could potentially explain why no significant differences were observed in healthy individuals in this study. Because the gut microbiota WAS significantly restored over time after appendectomy, appendectomy did not pose a health risk. It should be noted that this is only a hypothesis: since the timing of appendectomy in this study was unknown, no sound conclusions on the restorative tendency of the gut microbiota can be made from the results of this study.

Our results are in line with a study with a similar sample size (*N* = 1′097, wA *N* = 115) and data analysis plan (Goedert et al., 2014). In this specific study, a secondary data analysis was also performed on data from the American Gut Project, where, as in this study, subjects self-reported a history of appendectomy, and the timing of appendectomy was unknown, facing the same biases for generalization as this study. Curiously, in this respective study, age and BMI were also significantly higher in the appendectomy group (*p* < 0.001) (Goedert et al., 2014).

The restorative tendency of the gut microbiota, even in subjects who have undergone appendectomy, has been established in the literature (McGuinness et al., 2024). This phenomenon sheds new light on the association between appendectomy and the heightened risk of several different disease states (Rubin, 2019; Killinger et al., 2018; Kawanishi et al., 2017). Earlier work (Goedert et al., 2014), but also the original study (Cai et al., 2021) implies that the gut microbiota can recover after appendectomy with time, so that it does not differ in composition and functionality significantly from the gut microbiota of a person with an appendix. Because of the many unknown circumstances of appendectomy in this study, our results are not capable of confirming this hypothesis. However, curiously, our findings also lead in the same direction: the gut microbiota composition of healthy subjects with a history of appendectomy was distinguishable from that of non-healthy subjects with appendectomy but not indistinguishable from that of healthy subjects. These results support the idea that dysbiosis does not necessarily occur in all subjects after appendectomy, since the gut microbiota can normalize over time in certain individuals. Curiously, dysbiosis in the sense of a less diverse gut microbiota was predominantly found in non-healthy females after appendectomy (*p* < 0.01), which is in line with studies that associated appendectomy with various health risks in women, but not in men (Sawahata et al., 2017; Tzeng et al., 2015).

However, the gut microbiota’s restorative tendency, even in our study subjects who underwent appendectomy, was, on average, less healthy than their counterparts whose appendices remained in place. Among healthy subjects, only 11.2% had undergone appendectomy. In the general study population of 15′718 subjects, it was 16.6%. While 6.7% of the general study population was healthy, in the appendectomy group, it was only 4.2%, implying that the appendix does indeed contribute to one’s immune robustness (Laurin et al., 2011).

Human behavior is key to the maintenance of healthy gut microbiota (Quin and Gibson, 2020). Modifiable lifestyle factors are key to the state of the gut microbiota (Partula et al., 2019; Castonguay-Paradis et al., 2023). Our results imply that a healthy lifestyle, such as adequate sleep and fiber consumption, could potentially compensate for the effects of appendectomy on the gut microbiota, as seen in healthy subjects who underwent appendectomy in our study population.

The bidirectional positive relationship between adequate sleep and gut microbiota has been established in a great body of scientific work (Cavon et al., 2025; Wankhede et al., 2025), and our study supports the importance of adequate sleep quantity for diverse and functional gut microbiota (Matenchuk et al., 2020).

Our study had several strengths, including a relatively large sample size; high-quality, unbiased profiles of the microbiome; a restriction to adult individuals; careful exclusion of recent antibiotic use and specific medical conditions that might have altered the microbiota; and state-of-the-art statistical methods, including metrics of gut microbiota functionality and comparison based on a health state postulated to alter the gut microbiota.

## Limitations

The study’s critical limitation is that it presents observational data, which is retrospective in nature and relies on individual self-reporting, which inherently leads to a plethora of biases, especially selection and information biases. No data on presumed appendectomy are clinically validated, and therefore, interpretation has to be performed with great caution. Neither the timing nor the cause of the appendectomy is known because the medical records of the respective appendectomies are not available to researchers. This is a fundamental limitation for the interpretation of the study findings, as they suggest a potential restorative tendency of the gut microbiota.

Since the researchers did not have access to physician-confirmed diagnoses, their definition of health is substantially vague, especially because many subclinical disorders, such as insulin resistance and low-grade chronic inflammation, are not regarded as chronic illnesses by many individuals and do not require chronic medication, albeit having an impact on the gut microbiota. The term “healthy” is based on common exclusion criteria, not clinical evaluation; hence, possible misclassification bias could not be prevented in this study.

Unlike the original study, this study was cross-sectional and retrospective. Therefore, it is unclear whether the gut microbiota was restored over time, thereby diminishing any detrimental consequences of appendectomy, or confounding factors such as age were at play, reducing the importance of an appendectomy undergone in the distant past, e.g., in early infancy.

Another substantial limitation is that the cause of appendectomy is unknown, be it acute or chronic appendicitis, or even tumors. In the vast majority of cases—approximately 80%—appendectomy is performed to treat acute appendicitis. A smaller proportion, around 20%, are incidental prophylactic (incidental) appendectomies performed during other abdominal surgeries (Lee et al., 2019). Appendectomy due to appendiceal tumors is much less common, accounting for roughly 1% of cases (Furman et al., 2012). It is unclear whether the results were an effect of appendectomy or a preceding disease.

An important limitation is the selection bias of the database used, since it contains only the data of customers of the laboratory who paid for the gut microbiota analysis. This means that financial and sociological criteria were crucial in the assembly of the study population.

Dysbiosis is associated with a long list of disease states, especially chronic ones. Therefore, it is improbable that a healthy subject is suffering from dysbiosis. Since only limited medical data were accessible, especially in the form of laboratory parameters, it is unclear whether the applied exclusion criteria were justified for this specific healthy population.

Age, as a very important determinant of gut microbiota composition, significantly differed between HwA and HnoA. In the total study population, sex and BMI, as additional important determinants besides age, were significantly different between the wA and noA groups. This further limits the interpretation of the results of the comparison between these two respective groups. Both age and sex are significant predictors of gut microbiota variability (Dong et al., 2023; Li et al., 2024).

Another limitation is that all metadata of this study were self-reported via the respective lifestyle questionnaire, including age and appendectomy history. Since no medical or expert supervision of the data entries was conducted, the quality of the data is questionable.

## Data Availability

The data analyzed in this study was obtained from a proprietary human gut microbiome cohort generated by BIOMES NGS GmbH. The following restrictions apply: the underlying 16S rRNA gene sequencing data, including raw reads and ASV-level data, constitute core intellectual property and commercially sensitive information of BIOMES NGS GmbH and therefore cannot be made publicly available or shared with third parties outside the company. Requests to access these datasets should be directed to BIOMES NGS GmbH (attention: Carsten Krumbiegel, CTO) at science@biomes.world. Please note that due to the proprietary nature of the data and IP protection requirements, such requests cannot be granted.
