# Supplementary material for: The association between history of appendectomy and gut microbiota composition: a follow-up cross-sectional study
Source: Front Microbiol. 2026 Jan 7;16:1697138. doi: 10.3389/fmicb.2025.1697138 (PMC12819762; doi:10.3389/fmicb.2025.1697138)
Supplement: Supplementary file 1 [file Data_Sheet_1.pdf]

INTEST.pro Questionnaire

1. What is your gender?
   - [ ] Male
   - [ ] Female
   - [ ] Diverse

2. What year were you born?

3. What month were you born in?

4. How tall are you? (in cm)

5. How much do you weigh? (kg)

6. Have you gained or lost more than 5 kg compared to a year ago?
   - [ ] No
   - [ ] Gained
   - [ ] Lost
   - [ ] I don’t know

7. In which country are you at home?

8. Where do you live?
   - [ ] Large city
   - [ ] Medium-sized town
   - [ ] Small town
   - [ ] Village / rural community

9. What area do you live in?
   - [ ] Mainly mountains & mountain ranges
   - [ ] Predominantly flat land
   - [ ] Coast

10. Do you have any children?
    - [ ] Yes
    - [ ] No

11. Are you pregnant?
    - [ ] Yes
    - [ ] No

12. What trimester are you in?
    - [ ] First trimester
    - [ ] Second trimester
    - [ ] Third trimester

13. Are you currently breastfeeding?
    - [ ] Yes
    - [ ] No

14. Do you have any pets or are you often in contact with animals?
    - [ ] Yes
    - [ ] No

15. With what animals?
    - [ ] Dogs
    - [ ] Cats
    - [ ] Horses
    - [ ] Birds
    - [ ] Small animals (hamsters, rabbits, etc.)
    - [ ] Other

16. Do you smoke?
    - [ ] Yes
    - [ ] No

17. How many cigarettes do you smoke on average?
    - [ ] 1 to 5 cigarettes per day
    - [ ] 6 to 10 cigarettes per day
    - [ ] 11 to 20 cigarettes per day
    - [ ] More than 20 cigarettes per day

18. Why did you choose to take the test?
- ☐ I want to lose weight
- ☐ I sometimes suffer from intestinal and digestive problems
- ☐ I have permanent and severe digestive problems
- ☐ I am interested in intestinal flora-optimized nutrition
- ☐ I want to get fitter
- ☐ I want to strengthen my immune system.
- ☐ Other

19. How often do you travel to other countries?
- ☐ Once a year
- ☐ 2 to 3 times a year
- ☐ More than 3 times a year
- ☐ I don't travel

20. To which of these continents have you travelled in the last month?
- ☐ Africa
- ☐ Europe
- ☐ South Asia
- ☐ The Far East
- ☐ North America
- ☐ South America
- ☐ Australia
- ☐ I haven't been travelling in the last month

21. Here you can add comments on the topic of travel, e.g. particularly long stays abroad:

22. How would you describe your current job?
- ☐ Only sedentary work
- ☐ Sitting activity, sometimes also additional energy expenditure for walking or standing activities
- ☐ Predominantly walking or standing work
- ☐ People who do hard and strenuous physical work
- ☐ Not working at present

23. Do you often work at night (night shifts)?
- ☐ Yes
- ☐ No

24. Do you take any part in sports?
- ☐ Yes
- ☐ No

25. How often do you exercise on average?
- ☐ Less than twice a month
- ☐ Once every two weeks
- ☐ Once a week
- ☐ 1 to 2 times per week
- ☐ 3 to 4 times per week
- ☐ 5 to 6 times per week
- ☐ Every day

26. With what intensity do you mostly take part in sports?
- ☐ Low intensity
- ☐ Moderate intensity
- ☐ High intensity

27. How would you describe your general free time (without sports)?
- ☐ Almost inactive due to age/illness
- ☐ Mainly sitting/lying with few activities
- ☐ Normal daily burden
- ☐ Very active, with few rest periods

28. How long do you sleep on average?
- ☐ Less than 4 hours
- ☐ 4 to 5 hours
- ☐ 5 to 6 hours
- ☐ 6 to 7 hours
- ☐ 7 to 8 hours
- ☐ 8 hours or more

29. Do you feel refreshed after sleep?
- ☐ Yes
- ☐ No

30. Do you feel stressed out a lot?

31. The following question is very personal and may remain unanswered: Are you currently using any recreational drugs?
- ☐ Yes
- ☐ No

32. Which?

33. What type of diet do you keep to?
- ☐ Omnivorous
- ☐ Pescetarian
- ☐ Vegetarian
- ☐ Vegan

34. How often do you eat meat?
- [ ] Less than twice a month
- [ ] Once every two weeks
- [ ] Once a week
- [ ] 1 to 2 times per week
- [ ] 3 to 4 times per week
- [ ] 5 to 6 times per week
- [ ] Every day

35. Do you completely renounce certain types of meat?
- [ ] Yes
- [ ] No

36. What types of meat?
- [ ] Poultry
- [ ] Pig
- [ ] Beef
- [ ] Sheep, lamb, mutton
- [ ] Game meat
- [ ] Rabbit
- [ ] Other

37. How often do you eat fish?
- [ ] Never
- [ ] Less than twice a month
- [ ] Once every two weeks
- [ ] Once a week
- [ ] 1 to 2 times per week
- [ ] 3 to 4 times per week
- [ ] 5 to 6 times per week
- [ ] Every day

38. How often do you eat fruits and vegetables?
- [ ] Once a week
- [ ] 1 to 2 times per week
- [ ] 3 to 4 times per week
- [ ] 5 to 6 times per week
- [ ] Every day

39. How many portions of fruit & vegetables do you eat on average?

40. Do you have any special eating habits?

- ☐ Gluten Free
- ☐ Lactose free
- ☐ Histamine free
- ☐ No dairy products
- ☐ No eggs
- ☐ Ketogene diet
- ☐ Paleo
- ☐ Interval Fasting
- ☐ Low carbohydrates
- ☐ Low fats
- ☐ Low FODMAP
- ☐ SCD (Special Carbohydrate Diet)
- ☐ Protein Shakes
- ☐ Only raw vegetables
- ☐ Fructose-free
- ☐ Other
- ☐ Nothing applies

41. How often do you eat sweets?

- ☐ Never
- ☐ Less than twice a month
- ☐ Once every two weeks
- ☐ Once a week
- ☐ 1 to 2 times per week
- ☐ 3 to 4 times per week
- ☐ 5 to 6 times per week
- ☐ Every day

42. Which of the following foods do you eat on at least 3 of 7 days?

- ☐ Meat substitute products
- ☐ Finished products
- ☐ Food made from white flour or other refined grains
- ☐ Wholemeal products
- ☐ Pulses
- ☐ Nuts
- ☐ Nothing applies

43. Here you can write special remarks:

44. How much liquid do you drink on average per day?
- [ ] Less than a liter
- [ ] 1 to 1.5 liters
- [ ] 1.5 to 2 liters
- [ ] More than 2 liters

45. How often do you drink alcohol on average?
- [ ] Never
- [ ] Less than 5 times per month
- [ ] 1 to 2 times per week
- [ ] 3 to 4 times per week
- [ ] Every day

46. Which drinks do you have every day?
- [ ] Water
- [ ] Juice
- [ ] Soft drinks
- [ ] Tea
- [ ] Coffee
- [ ] Other

47. Are your soft drinks mainly sugary?
- [ ] Yes
- [ ] No

48. Do you drink your tea mainly with sugar?
- [ ] Yes
- [ ] No

49. Do you drink most of your coffee with sugar?
- [ ] Yes
- [ ] No

50. Do you take probiotics as a dietary supplement?
- [ ] No, never
- [ ] More than a year ago
- [ ] 6 to 12 months ago
- [ ] 3 to 6 months ago
- [ ] 1 to 3 months ago
- [ ] Less than a month ago
- [ ] At the moment, yes

51. Have you recently taken any of these BIOMES products?

- ☐ Alpha type
- ☐ Beta type
- ☐ Delta type
- ☐ Gamma type
- ☐ Epsilon type
- ☐ Zeta type
- ☐ DEFENSE.pro
- ☐ HISTA.pro (previously HISTAMIN.pro)
- ☐ LEAN.pro (previously DIET.pro)
- ☐ PREMIUM.pro
- ☐ RESTART.pro
- ☐ No

52. Do you often (at least 3 times a week) eat probiotic or fermented food?

- ☐ Kefir
- ☐ Yoghurt
- ☐ Sauerkraut (pickled cabbage)
- ☐ Pickled vegetables or fruit
- ☐ Kombucha
- ☐ Kimchi
- ☐ Miso
- ☐ Cheese
- ☐ Tempeh
- ☐ Natto
- ☐ Kvass, Togwa or Boza
- ☐ Cider
- ☐ Other
- ☐ No

53. Are you taking any other dietary supplements?

- ☐ Yes
- ☐ No

54. Do you take vitamins?

- ☐ Yes
- ☐ No

55. What vitamins?

- [ ] Vitamin A (retinol, carotenoids)
- [ ] Vitamin B complex
- [ ] Vitamin C (ascorbic acid)
- [ ] Vitamin D
- [ ] Vitamin E
- [ ] Vitamin K
- [ ] I don't know exactly

56. Do you take trace elements or minerals?

- [ ] Yes
- [ ] No

57. Which minerals/trace elements?

- [ ] Calcium
- [ ] Iron
- [ ] Iodine
- [ ] Magnesium
- [ ] Zinc
- [ ] Selenium
- [ ] I don't know exactly

58. Do you take other types of supplements?

- [ ] Yes
- [ ] No

59. What other types of food supplements?

- [ ] Amino acids
- [ ] Hyaluronic acid
- [ ] Omega-3 fatty acids
- [ ] OPC or other bioflavonoids
- [ ] I don't know exactly
- [ ] Other

60. How would you describe your current well-being?

61. How would you assess your current state of health?

62. Do you have any allergies or intolerances?

- [ ] Yes
- [ ] No

63. What allergies or intolerances do you have?

64. When was the last time you took antibiotics?
- [ ] No, never
- [ ] More than a year ago
- [ ] 6 to 12 months ago
- [ ] 3 to 6 months ago
- [ ] 1 to 3 months ago
- [ ] Less than a month ago
- [ ] At the moment, yes

65. Do you suffer from migraine?
- [ ] Yes, I think so
- [ ] Yes, diagnosed
- [ ] No, but frequent headaches
- [ ] No

66. How often have you had migraines in the last three months?
- [ ] Less than 3 times
- [ ] Between 3 and 9 times
- [ ] More often than 10 times

67. How often do you suffer from colds?
- [ ] Once a year or less
- [ ] 2 to 3 times a year
- [ ] 4 to 5 times a year
- [ ] More than 5 times a year

68. Are you currently suffering from one or more of these digestive disorders?
- [ ] Diarrhea
- [ ] Constipation
- [ ] Flatulence
- [ ] Abdominal pain / abdominal cramps
- [ ] Irritable bowel syndrome
- [ ] No

69. Have you had diarrhea for two months or longer?
- [ ] Yes
- [ ] No

70. Have you been constipated for three months or more?
- [ ] Yes
- [ ] No

71. How often do you have bowel movements on average?
- [ ] More than 3 times a day
- [ ] 2 to 3 times per day
- [ ] Every day
- [ ] Once every two days
- [ ] 3 times or less per week
- [ ] Very irregular

72. How would you describe the consistency of your stool?
- [ ] Type 1
- [ ] Type 2
- [ ] Type 3
- [ ] Type 4
- [ ] Type 5
- [ ] Type 6
- [ ] Type 7
- [ ] I don't remember.

73. Have you had recurrent abdominal pain on at least one day a week in the past three months?
- [ ] Yes
- [ ] No

74. Is this related to at least one of the following conditions (multiple selection possible)?
- [ ] In connection with the bowel movement
- [ ] Associated with an altered frequency of bowel movement
- [ ] Combined with a modified stool shape
- [ ] No

75. Did this happen 6 months ago or longer?
- [ ] Yes
- [ ] No

76. Where would you assign the stool sample based on the picture/scale?
- [ ] More than one quarter (25%) of bowel movements according to Bristol type 1-2 and less than one quarter (25%) according to Bristol type 6-7
- [ ] More than a quarter (25%) of bowel movements according to Bristol type 6-7 and less than a quarter (25%) according to Bristol type 1-2.
- [ ] More than one quarter (25%) of bowel movement according to Bristol Type 1-2 and more than one quarter (25%) according to Bristol Type 6-7
- [ ] Intestinal movement which is not precisely classified into one of the types mentioned above.

77. Has your appendix been removed?
- [ ] Yes
- [ ] No

78. Are you currently suffering from any illnesses?

- [ ] Yes
- [ ] No

79. What diseases and/or complaints do you have?

80. Do you have any other comment?

81. Are you currently taking any medication?

- [ ] Yes
- [ ] No

82. If yes, what for?

- [ ] (Sugar) metabolism and digestion
- [ ] Infectious diseases (anti-infectives), and medicines against parasites
- [ ] Nervous system, locomotor system
- [ ] Heart, circulation, blood & blood-forming organs
- [ ] Respiratory tract (or respiratory organs)
- [ ] Skin
- [ ] Eye & ear
- [ ] Sex hormones and the urogenital system
- [ ] Hormone preparations WITHOUT sex hormones and insulin
- [ ] Tumor therapy and immunomodulating therapies
